# Development of a bioeconomy monitoring framework for the European Union: An integrative and collaborative approach

**DOI:** 10.1016/j.nbt.2020.06.001

**Published:** 2020-11-25

**Authors:** Nicolas Robert, Jacopo Giuntoli, Rita Araujo, Marios Avraamides, Elisabetta Balzi, José I. Barredo, Bettina Baruth, William Becker, Maria Teresa Borzacchiello, Claudia Bulgheroni, Andrea Camia, Gianluca Fiore, Marco Follador, Patricia Gurria, Alessandra la Notte, Maria Lusser, Luisa Marelli, Robert M’Barek, Claudia Parisi, George Philippidis, Tévécia Ronzon, Serenella Sala, Javier Sanchez Lopez, Sarah Mubareka

**Affiliations:** a European Commission, Joint Research Centre (JRC), Italy; b Formerly with the European Commission, Joint Research Centre (JRC), Italy; c European Commission, Joint Research Centre (JRC), Spain; d European Commission, Joint Research Centre (JRC), Belgium; e Formerly with the European Commission, Joint Research Centre (JRC), Spain

**Keywords:** CAP, Common Agricultural Policy, CFP, Common Fisheries Policy, EC, European Commission, ES, ecosystem services, EU, European Union, FAO, Food and Agriculture Organization of the United Nations, IBF, International Bioeconomy Forum, ISBWG, International Sustainable Bioeconomy Working Group, JRC, EC Joint Research Centre, LCA, Life Cycle Assessment, MAES, Mapping and Assessment of Ecosystem Services, MS, Member States, NCA, natural capital accounting, SDG, Sustainable Development Goal, Monitoring framework, European Union, EU bioeconomy strategy, Sustainable circular economy, Sustainable development goals, Ecosystem, Climate change, Environment, Biomass

## Abstract

The EU Bioeconomy Strategy, updated in 2018, in its Action Plan pledges an EU-wide, internationally coherent monitoring system to track economic, environmental and social progress towards a sustainable bioeconomy. This paper presents the approach taken by the European Commission’s (EC) Joint Research Centre (JRC) to develop such a system. To accomplish this, we capitalise on (1) the experiences of existing indicator frameworks; (2) stakeholder knowledge and expectations; and (3) national experiences and expertise. This approach is taken to ensure coherence with other bioeconomy-related European monitoring frameworks, the usefulness for decision-making and consistency with national and international initiatives to monitor the bioeconomy. We develop a conceptual framework, based on the definition of a sustainable bioeconomy as stated in the Strategy, for a holistic analysis of the trends in the bioeconomy sectors, following the three pillars of sustainability (economy, society and environment). From this conceptual framework, we derive an implementation framework that aims to highlight the synergies and trade-offs across the five objectives of the Bioeconomy Strategy in a coherent way. The EU Bioeconomy Monitoring System will be publicly available on the web platform of the EC Knowledge Centre for Bioeconomy.

## Highlights

• An EU-wide internationally coherent system to monitor the bioeconomy is described.
• The system will provide information on the sustainability of the bioeconomy.
• A multi-dimensional and comprehensive framework is required.
• EU and international experts have contributed to the design of the system.
• The system will be published through the EC’s Knowledge Centre for Bioeconomy.

## Graphical abstract

Integrative and collaborative process adopted to design the EU Bioeconomy Monitoring System

**Figure:**
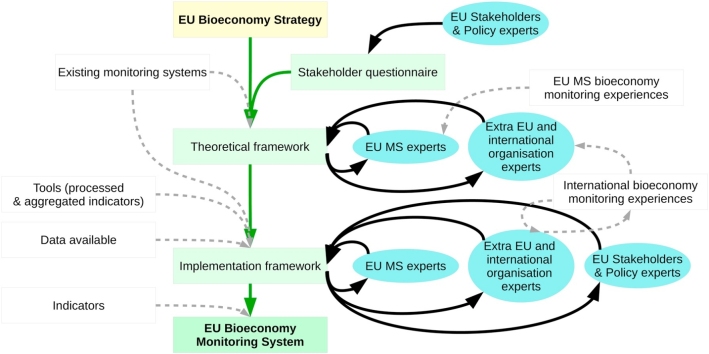

## Introduction

In the context of global challenges such as climate change, ecosystem degradation, biodiversity loss, growing population, and increasing consumption of resources, the European Union (EU) aims to pursue sustainable development and to guarantee fair and inclusive prosperity within the ecological boundaries of the planet [1,2]. The shift to a sustainable bioeconomy (see definition in Box 1) is arguably relevant to the success of many EU policies and is expected to contribute to the overall objectives and specific initiatives of the European Green Deal [3]. The EU Bioeconomy Strategy (hereafter, also the “Strategy”), operates within a complex existing policy context, which includes sectorial policies such as the Common Agricultural Policy (CAP) [4], the Common Fisheries Policy (CFP) [5], the New Industrial Strategy for Europe [6]; as well as cross-sectorial policies such as the European strategic long-term vision for a prosperous, modern, competitive and climate neutral economy [7], the 2030 Climate and Energy Framework (where the bioeconomy is among the seven strategic building blocks of the EU long-term vision to reach climate-neutrality by 2050), the EU Biodiversity Strategy [8], Europe’s strategy for research and innovation, the European action for sustainability [9], the EU trade policy, the new Circular Economy Action Plan [10] and many more [11]. Being at the confluence, the Bioeconomy Strategy with its five objectives (see first column of Table 1) is regarded by EU policy makers as pivotal to improving the coherence between those policies aiming to lead the EU society towards more sustainable pathways by decoupling economic growth and environmental impacts, by lowering greenhouse gas emissions and restoring ecosystems while providing jobs and services.

Box 1 **Sustainable & Circular: Bioeconomy the European way ([1], p. 1).**

“The bioeconomy covers all sectors and systems that rely on biological resources (animals, plants, micro-organisms and derived biomass, including organic waste), their functions and principles. It includes and interlinks: land and marine ecosystems and the services they provide; all primary production sectors that use and produce biological resources (agriculture, forestry, fisheries and aquaculture); and all economic and industrial sectors that use biological resources and processes to produce food, feed, bio-based products, energy and services. To be successful, the European bioeconomy needs to have sustainability and circularity at its heart. This will drive the renewal of our industries, the modernisation of our primary production systems, the protection of the environment and will enhance biodiversity.”

Alt-text: Box 1

**Table 1:** Objectives of the 2018 EU Bioeconomy Strategy and the main criteria to monitor its effectiveness. The mapping to SDGs is carried out comparing key components/criteria of the EU Bioeconomy framework to UN SDG targets.

### Knowledge as a basis for the bioeconomy development

Being a part of complex socio-economic and environmental systems, it is difficult to foresee all of the direct and indirect impacts of the bioeconomy, and trade-offs are expected. Moreover, the impacts are conditioned by the pathways that individual countries and regions follow. There is therefore a need for comprehensive, reliable and comparable information on the bioeconomy and its progress to support decision making across sectors and across the EU territory at different scales [[12], [13], [14]]. In this context, the European Commission (EC) has committed to provide reliable and harmonised data, information, and knowledge concerning the bioeconomy to policy makers and other stakeholders (see Action Plan, [1], p. 13).

The EC Joint Research Centre (JRC) launched a long-term study in 2015 in order to provide a sound scientific basis for EC policy making. The aim of the work is to provide data, processed information, models and analysis on EU and global biomass supply, demand and its sustainability [15]. The first results of the comprehensive evaluation of biomass supply, uses and flows were released in 2018 [16]. They show the status and trends in all primary sectors (agriculture, forestry, fisheries and aquaculture).

The EC has invested in managing knowledge about the bioeconomy and its impacts since the release of the 2012 EU Bioeconomy Strategy [17]. In 2013, the Bioeconomy Information System and Observatory (BISO) were established by the JRC with the purpose of structuring and facilitating access to information. This included information on the environmental performance of some bioeconomy value chains [18], as well as on socio-economic indicators [19], bio-based industries [20], and forward-looking scenarios of the bioeconomy [21]. These efforts were merged into the EC’s Knowledge Centre for Bioeconomy in 2017. This Centre is coordinated by the JRC and manages knowledge and expertise from inside and outside the EC by facilitating knowledge sharing between experts, scientists and policy makers and by providing a dissemination platform [22]. The Knowledge Centre for Bioeconomy will host the Bioeconomy Monitoring System on its online platform as foreseen in the updated EU Bioeconomy Strategy and Action Plan [1].

### The need for a monitoring system

The goal of monitoring systems is to monitor change, and in the case of the EU bioeconomy, progress. ‘Progress’ indicates the advancement towards an established goal or an improved condition. Policy makers expressed the need for a transparent and comprehensive assessment of the status and progress of the bioeconomy to legislate and take decisions [23,24]. Hence one of the 2018 EU Bioeconomy Strategy actions foresees the development of an EU-wide, internationally coherent monitoring system to track economic, social and environmental progress towards a circular and sustainable bioeconomy. The JRC is leading this action, in collaboration with several Commission Services and stakeholders (see Action Plan in [1]).

The monitoring system, an essential tool for reflexive governance [25], will facilitate the evaluation of progress towards the objectives of the Strategy and support the identification of areas in need of policy intervention. Furthermore, it will provide the tools for cross-sectorial and therefore cross-policy assessments. In addition, the system will highlight synergies and trade-offs across multiple scales and levels: geographical (global, EU, national and regional); between the five Strategy objectives; across pillars of sustainability; and across economic sectors. Given the multi-faceted nature of the bioeconomy, a clear conceptual framework is seen by many scholars as being necessary [12,[26], [27], [28]].

In this paper, we present the methodological steps taken by the JRC (1) to define a monitoring framework flexible enough to inform a wide-range of stakeholders for different purposes; (2) to interact with national and international organisations to ensure the coherence between the different monitoring schemes; (3) to implement the EU monitoring framework into an operational system; and (4) the way forward.

### From the definition and objectives of the EU bioeconomy to the conceptual framework of the monitoring system

First, we delineate the thematic perimeter and key features of the bioeconomy to ensure that all relevant facets are represented. Bioeconomy may be defined in various ways: in the EU case, the definition given in the Strategy (see Box 1) is broad and encompassing. Secondly, to ensure the usefulness of the system, stakeholders’ expectations should be taken into account to represent available information in a way that meets their needs while being scientifically sound. Thirdly, other bioeconomy or related monitoring systems (both existing or under development) are reviewed in order to ensure the coherence between national and international systems, as well as to limit the reporting burden by using existing information as much as possible.

### Evaluating the bioeconomy from three perspectives

Our work departs from the definition used in the Strategy, which uses several keywords that are guidelines to define the framework. From the definition, we elaborate a three-dimension conceptual framework as follows.

According to the Strategy, the bioeconomy covers the primary sectors (agriculture, forestry, fisheries and aquaculture) and the ecosystems supplying primary goods and services, which constitute the foundation of the bioeconomy. These sectors are clearly identified in statistics and targeted by national and/or EU policies. Therefore, the primary sectors constitute a first dimension that complies with sectorial knowledge.

The bioeconomy covers economic activities performed in several sectors of the countries’ economies as well as the part of the economic systems that rely on “biological resources […], their functions and principles”. This indicates that the monitoring framework must cover the whole bioeconomy value chains from the supply of biomass and other goods and services by the primary sectors, to the transformation and final use of these goods and services as well as the possible reuse and recycling of the biomass. Therefore, the bioeconomy value chains constitute the second dimension of the conceptual framework.

The Strategy also specifies that “all economic and industrial sectors that use biological resources and processes to produce food, feed, bio-based products, energy and services” are covered by the definition of bioeconomy. This means that the use of biotechnology (in the processes) is within the scope of the new Strategy, independently from the type of feedstock used, with the exception of biomedicines and health biotechnology, which are explicitly excluded as stated in the EU Bioeconomy Strategy [1].

The definition in the Strategy ends with a normative requirement for the bioeconomy to be successful: “the European bioeconomy needs to have sustainability […] at its heart”. Therefore, monitoring the progress towards a bioeconomy requires a comprehensive evaluation across the three sustainability pillars: economic, social and environmental. This constitutes a third dimension to be monitored.

**Fig. 1:**
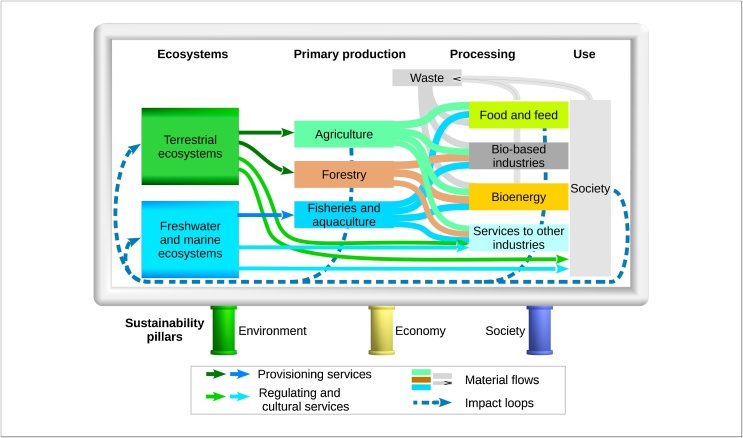
Theoretical framework to monitor the sustainable circular bioeconomy.

The three dimensions of the definition as mentioned above represent the structure of a conceptual framework (Fig. 1) which serves as a basis to identify data needs for the monitoring system. It is also of interest to verify that the monitoring system does not lack key indicators and that it uses adequate weights for the different aspects of the bioeconomy.

### Understanding users’ expectations

A survey on users’ expectations from an EU Bioeconomy Monitoring System was conducted in April-May 2019 using a snowball sampling technique [29]. We collected feedback from 76 participants, mostly from governmental institutions, covering 18 EU Member States (MS): Austria, Croatia, Denmark, Estonia, Finland, France, Germany, Ireland, Italy, Latvia, Lithuania, Poland, Portugal, Slovakia, Slovenia, Spain, Sweden and the Netherlands. The feedback received indicated that the System is expected to provide information at both EU and MS levels, as well as on rural and coastal areas, and should convey knowledge about all sectors related to the bioeconomy. Users expressed their need for time series of indicators to analyse trends. They also wished for analytical knowledge, in particular of synergies and trade-offs related to the development of the bioeconomy to be within the monitoring system.

Participants to the survey wanted to use the monitoring system to compare MS performance in different sectors; to follow rural, coastal and regional trends to support the deployment of bioeconomies at local level. The main goals of the potential users were stated as (1) prioritisation of actions and (2) to inform stakeholders. The user requirements will be further refined through workshops and dialogues with users from the European institutions, and national and international experts.

### Collaboration with other national and international bioeconomy monitoring initiatives

Maintaining exchanges with national and international organisations is important to ensure that the EU Bioeconomy Monitoring System is up-to-date with similar concepts and definitions in the worldwide arena. It is worth noting that the concept of bioeconomy varies between different initiatives and that these concepts may change over time, just as the definition of the EU bioeconomy evolved from the 2012 to the 2018 Strategy. As the Organisation for Economic Co-operation and Development (OECD) reports, the bioeconomy has “grown from a biotechnology-centric vision to an economic activity that spreads across several key sectors and policy families: agriculture and forestry, fisheries and aquaculture, food, trade, waste management and industry” [30].

Joining the EU and international perspectives, the JRC, at the forefront of the EC’s task to establish an EU Bioeconomy Monitoring System, is co-chairing the Bioeconomy Indicator Working Group of the International Bioeconomy Forum (IBF) with the Food and Agriculture Organization of the United Nations (FAO) to prepare guidelines for the monitoring of the bioeconomy. To ensure compatibility with the international arena, the JRC is following the International Sustainable Bioeconomy Working Group (ISBWG) structure of 10 aspirational principles and 24 criteria [13,31] in its implementation framework by mapping them to the five EU Bioeconomy objectives.

**Fig. 2:**
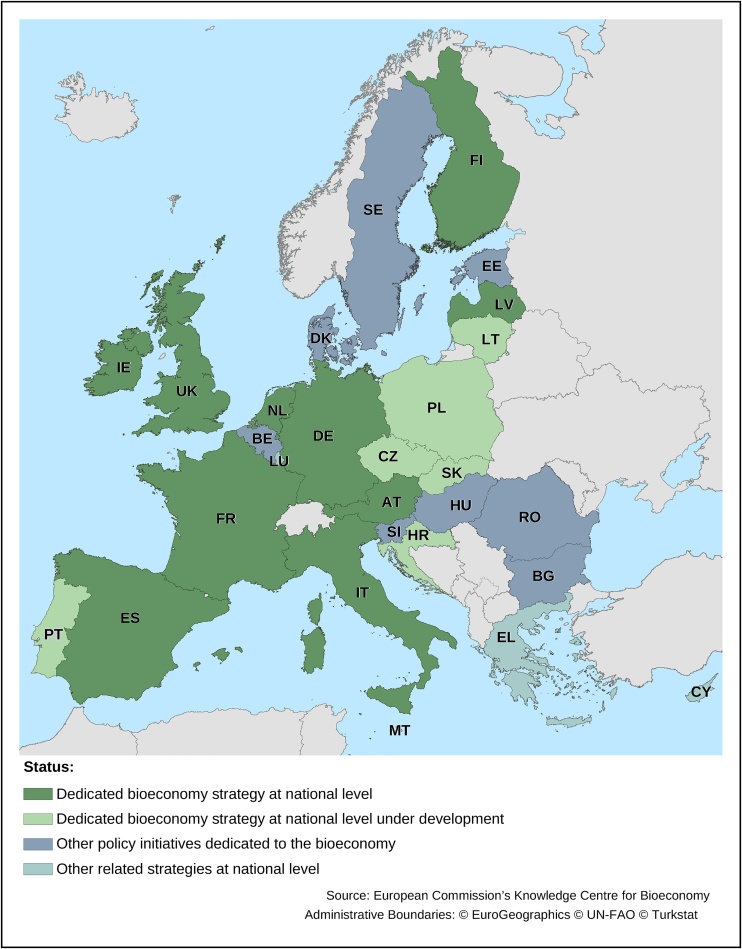
Strategies and other policy initiatives dedicated to the bioeconomy in the EU Member States (Status of as of November 2019).

The JRC is following a bi-directional and mutual-learning approach with MSs to develop the EU Bioeconomy Monitoring System. Several EU MSs have released their own national bioeconomy strategies [32]. Pioneers were Germany in 2010 and Finland in 2014, followed by Spain in 2016, France, Italy and Latvia in 2017, Ireland, the Netherlands and the United Kingdom (The UK was part of the EU at the time of this analysis) in 2018 and Austria in 2019 (Fig. 2). Eastern European countries launched the BIOEAST initiative in November 2016 to develop a common roadmap and vision for the macro region and to support the development of national circular and bioeconomy strategies. Some of these countries are developing monitoring frameworks to assess progress in their bioeconomy. Given the variety of environmental and socio-economic contexts and the specific objectives of the national strategies, these frameworks differ. However, these national initiatives are an additional source of inspiration to setup the EU-wide Bioeconomy Monitoring System. Reversely, some MSs, inter alia Ireland [33] and Italy [34], explicitly state in their action plans the willingness to liaise and be consistent with the EU Commission on the EU-wide Bioeconomy Monitoring System.

The sectors and aspects covered in national bioeconomy strategies vary. Some countries such as Finland started with a focus on the socio-economic dimensions of the bioeconomy [35], while additional metrics to assess more comprehensively the impacts of the national bioeconomy will be considered in the future. Other national frameworks cover the biophysical and technological dimensions: in Germany, the monitoring framework is structured along 3 topic areas: (1) resources and their sustainability; (2) economic effects and economic development of the bio-economy, and (3) systemic monitoring, integrating data, indicators and models to provide a systemic, holistic insight into the bioeconomy [36]. The monitoring system in Italy relies currently on 8 areas: biomass availability, productive and employment structure, human capacity, innovation, investment, demographics and markets. Furthermore, the Italian Strategy considers separately an additional set of sustainability indicators structured along 5 environmental and social objectives which are in line with the EU Strategy objectives [34].

Several efforts have been carried out, or are ongoing, to select or define indicators. Lier et al. [37] conducted a study whereby the indicators that were prioritised by MSs at the time of writing were compared within the context of the MontBioeco project [38]. The MSs were approached through the Standing Committee on Agricultural Research Bioeconomy Strategic Working Group (SCAR-BSW) members. Thirteen countries, represented by different ministries, responded. Respondents confirmed that agriculture, aquaculture, fisheries, forestry, and food industry were always considered as part of the bioeconomy. Transport, water purification and distribution and construction were considered by some countries as being partially or not at all part of the bioeconomy, and the bio-based shares in the pharmaceutical industry and chemical industry processes was difficult to calculate.

The Horizon 2020 research project BioMonitor aims to establish a sustainable and robust framework to monitor the bioeconomy and its various impacts in EU Member States. It focuses on the elaboration of a comprehensive database and statistics on bio-based activities (including natural-resource-based activities, conventional bio-based activities and novel activities). During a first workshop, participating stakeholders indicated that the monitoring system developed in the project should track innovation, evaluate the capacity of the bioeconomy to mitigate climate change, inform about the bioeconomy, assess trade-offs in the transition from a fossil to a bio-based economy, and support policy decisions [39].

The on-going efforts summarised above illustrate the relevance of mutual learning, and of ensuring the coherence across scales, so that the EU Bioeconomy Monitoring System reflects national priorities and indicators, and that national initiatives benefit from a coherent EU-wide framework.

### Mobilising expertise in the EU institutions and Member States

In light of the number of initiatives mentioned above, numerous researchers and stakeholders in national and international organisations have gained expertise on the bioeconomy. The Knowledge Centre for Bioeconomy supports the mobilisation of this expertise, which is of interest to support the development of the EU Bioeconomy Monitoring System. In particular, it includes a Community of Practice, a network of EC scientists, policy makers and other experts who share information and perspectives on the bioeconomy, either in person or virtually [22].

Within this context, the Knowledge Centre for Bioeconomy organised two workshops in November 2018 and June 2019 to complement the knowledge of the JRC researchers on ongoing efforts to monitor the bioeconomy, to get feedback on draft versions of the EU monitoring framework and to establish a first list of indicators [40]. The interaction with stakeholders and scientists will continue thanks to the organisation of other workshops and the publication of working documents open for comments.

## Implementation of an EU Bioeconomy Monitoring System

Based on inter-disciplinary knowledge and skills in the EC and on the interaction with national and international experts, once the conceptual groundwork was laid down, the JRC began the implementation of a first framework to monitor the EU Bioeconomy. In this section, we develop the logic underlying this implementation framework.

### Implementation framework

The EU Bioeconomy objectives provide a broad vision for a sustainable bioeconomy. When implementing the conceptual framework, these objectives were disaggregated into normative criteria taking inspiration from the work by Bracco et al. [13], and then reframed and refined into key components tailored to the EU specificities. The criteria capture the vision that a sustainable EU bioeconomy should contribute to moving towards the Sustainable Development Goals (SDG), reaching climate-neutrality, promoting a circular economy, and encouraging a transition towards sustainable food, farming and fishing systems as well as towards sustainable forestry and the development of bio-based sectors. Preserving Europe’s natural capital for future generations, restoring ecosystems and enhancing their functions while conserving biodiversity are also core pillars of the Strategy. Furthermore, a sustainable and circular bioeconomy should create economic opportunities for rural, coastal and urban communities through local bio-based innovation, the integration of primary producers in value chains, the diversification of supply chains and the modernisation of EU industries. Finally, a sustainable EU bioeconomy must look beyond EU borders and promote sustainable trade conditions, promoting social fairness, economic growth, and environmental protection within trading countries.

Table 1 introduces the proposed framework highlighting the link with SDGs. Additional details and in-depth description of the framework can be found in [31]. The criteria provide a guideline for which indicators will be selected, with a preference for established indicators that are already used in other monitoring processes.

We identified two major sources of indicators covering many of the dimensions of the implementation framework. The first is the system to monitor the progress towards the UN SDGs at global and EU levels [[41], [42], [43]]. This system shares some critical characteristics with the EU Bioeconomy Monitoring System: they both need to capture aspects of complex interconnected systems, involving a large number of sectors at different geographical levels. Since the scope of the SDGs is wider than that of the bioeconomy, the seventeen SDGs and their specific targets constitute a “checklist” to assess the full coverage of sustainability aspects within the bioeconomy monitoring framework (see e.g [44,45].). In some cases, where the bioeconomy is the principal driver, the indicators can be similar, for example in the case of SDG targets 2.4 (Ensure sustainable food production systems) or 15.2 (Promotion of sustainable forest management). In other cases, in which the bioeconomy can contribute to meet the targets such as the SDG targets 7.2 (Increase in the share of renewable energy in the global energy mix) and 8.2 (Achieve higher levels of economic productivity), the SDG indicators can be used as references.

The second source of indicators is the monitoring of EU sectoral policies dealing with primary productions. For example a Common Monitoring and Evaluation Framework (CMEF) [46] was established by the EC in 2014 to assess the CAP. The EU CFP [5] is assessed by the Scientific, Technical and Economic Committee for Fisheries (STECF) using a set of environmental, economic and social indicators structured in CFP performance monitoring reports [47]. European forests, their function and services as well as the forest-based sector have been monitored since the 1990s in the context of Forest Europe [48]. These frameworks are all designed to assess the sustainability of the specific sectors they are directed towards. They are widely accepted by stakeholders and provide documented and replicable information. Therefore, although they differ in contents and approaches, they constitute a major source of indicators for the EU Bioeconomy Monitoring System.

Additionally, the monitoring of the objectives of the EU Bioeconomy Strategy is aligned with some specific monitoring systems. The FAO’s Food Security indicators and its four key components: Availability, Access, Utilisation, and Stability [49] were used to structure the assessment of objective 1.

The first criterion “Ecosystem condition and biodiversity are maintained or enhanced” of objective 2 is documented using indicators from the EU’s Mapping and Assessment of Ecosystem Services (MAES) initiative [50]. This initiative aims at supporting the implementation of the Biodiversity Strategy to 2020 and the new Strategy to 2030. In its fifth report [50], MAES provided an integrated analytical framework and set of (spatially-explicit) indicators for mapping and assessing the condition of ecosystems in the EU.

Objective 4: “Mitigating and adapting to climate change” is evaluated using the concepts from the Intergovernmental Panel on Climate Change (IPCC [51]) on mitigation and adaptation. Finally, tools to monitor Objectives 3 and 5 derive from different sources and make use of techniques such as macroeconomic analysis or life cycle analysis.

### Mapping indicators within the monitoring system

**Fig. 3:**
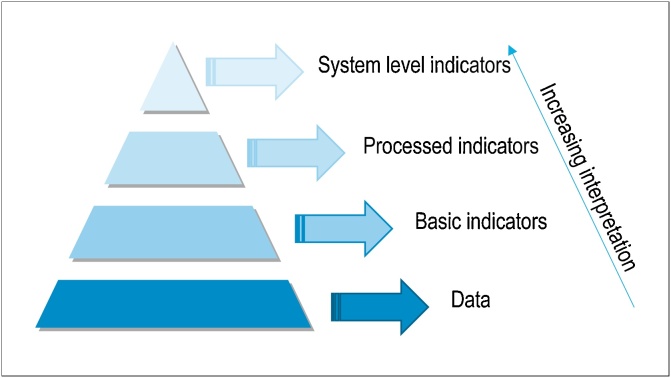
Illustration of the pyramid of information applied to the conceptual framework.

When mapping indicators to normative criteria, it becomes apparent that different types of indicators are required to answer specific questions. There are therefore, necessarily, different levels of indicators within the EU Bioeconomy Monitoring System (Fig. 3, [52]). At the foundation of the pyramid are underlying statistical data that can be measured, followed by three tiers of indicators differing in complexity, and thus increasingly subject to interpretation. The indicators are chosen based on their suitability to address the particular normative criteria that needs to be assessed. In some cases it is appropriate to use basic indicators whereas in other cases it is appropriate to use processed or system level indicators. Sometimes, the system level indicators make use of basic or processed indicators, but not always.

#### Basic indicators and data

The monitoring system will make use of existing and internationally recognised indicators to limit the reporting burden. Existing frameworks provide information through reporting systems that are not necessarily adapted to the bioeconomy, but whose indicators, at their most basic level, may be relevant to the bioeconomy. They are mapped within the conceptual framework described in section 2, i.e. within (1) the biomass source (i.e. agriculture, forest, fisheries and aquaculture as well as waste); (2) the step in the value chain, from the primary production to the final product, recycling and end-of-life; and (3) three sustainability pillars: economic, social and environmental, acknowledging the corresponding SDG targets.

For these basic indicators, criteria such as relevance, coverage, frequency and timeliness are important to consider (see e.g. [53]). Experts from various bioeconomy fields reviewed candidate indicators and created a “passport” containing the characteristics of each indicator (see Table 5 in [31]). The passport includes information such as the source of the indicator, the availability of recent and frequent estimates, the geographical coverage, the accessibility and the current use in other monitoring systems. These passports, together with expert knowledge, are used to select the most appropriate indicators. The mapping of these indicators onto the framework of the monitoring system highlights the main data gaps. During this mapping phase, criteria and key components of the framework are detailed. Gaps are filled with placeholders, i.e. a description of the characteristics of the expected indicator, to pinpoint the need for further research.

#### Processed indicators for harmonised, coherent and comprehensive information

The primary information given by basic indicators is normalised into processed indicators to provide a clearer, while still comprehensive, image of the progress towards the bioeconomy (see Fig. 3). The need for processed indicators arises for different reasons. Here we introduce three examples of processing that may be applied within the EU Bioeconomy Monitoring System (see some examples in [[54], [55], [56]]).

The monitoring system is designed such that it should combine numerous indicators about different facets of the bioeconomy from different sources. These indicators use different units and definitions depending on their sources, the sector they apply to, and qualify some characteristics which are hardly comparable (such as biodiversity and employment). In this section, we detail some techniques used to shape the EU Bioeconomy Monitoring System to provide a comprehensive insight.

The biomass is the main good produced and transformed in the bioeconomy. The monitoring system should provide an overall picture of the use of this resource and evaluate bioeconomy development opportunities–in line with the Bioeconomy objectives– to answer questions related to food security, to the sustainable management of natural resources, the competitiveness of the EU industries, the carbon flows and the dependence on renewable and non-renewable biomass resources. Therefore, the biomass supply and its flow through the economy must be represented in a coherent way. The biomass comes from a variety of sources: agriculture, forestry, fisheries and aquaculture as well as waste. Statistics on these biomasses and the products are usually not comparable. To align them, the JRC developed methods to harmonise the data and coefficients to estimate quantities in dry matter [16,54]. This allows for a comparison across biomass sources and sectors. However, the choice of the unit (e.g. mass of dry or fresh matter, monetary value) changes the perspective on the primary sectors and their relative importance. Therefore, different harmonisation units might be selected depending on the aspect to be represented in the framework.

To assess the fifth objective of the Strategy and to evaluate the contribution of the bioeconomy to the whole economy [57], the monitoring framework should contain indicators that cut across the economic sectors. Official statistics offer a wide variety of fit-for-purpose indicators (e.g. employment, value added, and trade). However, they are reported in different statistics according to specific definitions and according to official classifications of activity sectors or products that sometimes mix bio-based activities with non-bio-based ones. Methodologies were developed to harmonise the basic indicators and, for sectors only partially included in the bioeconomy, to extract the share of bioeconomy-related activities. They consist in calculating processed indicators using basic indicators observed at the level of the bioeconomy activity sectors. The indicators could be derived from input-output analyses (e.g [58].), from social accounting matrices (e.g [59,60].) or the combination of multiple economic statistics (such as the National Accounts, the Structural Business Statistics or the Labour Force Survey) with expert information (e.g [55,56,61].). Therefore, they embed a higher level of interpretation than basic indicators.

With the development of the bioeconomy, the EU will produce and consume more biomass. The EU’s own biomass resources will meet part of the demand although these ambitious targets will also require reliable and sustained access to global suppliers. The access to third-country trade raises concerns related to cropland footprint and emissions from direct and indirect land use change, as well as to changes in the pressure on natural resources and potential demand/supply conflicts, which in turn will require careful consideration of the possible trade-offs. The first and the third objectives of the Strategy include a component on trade. The corresponding normative criteria aim to assess whether the bioeconomy promotes sustainable trade of biomass for food and non-food uses. This is analysed through a combination of statistics and modelling to calculate processed indicators of the impacts the European trade on the partner countries [62,63].

#### System-level indicators to provide an overview

System-level indicators are those that require a higher level of value-judgement in their compilation given the higher level of complexity of the questions the indicators are addressing. We present below three methods that can provide relevant system level information on the bioeconomy.

To bring together the information on the state of ecosystems, the supply of biomass and other ecosystem services (ESs) and the economic activities, some system level indicators can be calculated based on natural capital accounting (NCA). This satellite account system is meant to integrate official economic accounts by using their same framework and methodological rules [64,65]. It therefore guarantees consistency with tools and models used by economists and thus allows integrated analysis and to analyse the role of the bioeconomy in the total economy of a country. The methodology developed in the knowledge innovation project on an integrated system of natural capital and ecosystem services accounting (KIP-INCA) can inspire the preparation of some bioeconomy indicators. In particular, information on the actual flow of ecosystem services (i.e. the flow supplied by ecosystem types and used by economic sectors and households) to the economy can be a starting point to characterize the bioeconomy. It can support the analysis of a wide range of issues, such as assessing whether the demand from economic sectors is met, how sustainably ESs are used, and how ES status and flows change over time [[66], [67], [68], [69]].

A method to elaborate indicators capable of unveiling causality links and trade-offs, embracing multiple dimensions (all life cycle stages along supply chains and different types of impacts) is Life Cycle Assessment (LCA). This method holistically addresses production and consumption systems, spillover and transboundary effects, and, relating all emissions and impacts to a product or function provided, is fit for sustainability assessment [70]. It is the basis for the calculation of the EU product environmental footprint [17], which covers 16 impact categories (such as climate change, ozone depletion, human toxicity particulate matter, acidification, land use, and water use). This approach makes it possible to evaluate the environmental impacts of the development of the EU bioeconomy in the EU and in partner countries. Furthermore, bioeconomy-related environmental hotspots may be identified through this methodology [13,71].

In general, the LCA characterises impacts based on an inventory of emissions and of resource use. The underpinning models to estimate those emissions are either very detailed and product-specific (as in process-based LCA) or associated to sectors or product groups (as in environmentally extended input-output analysis). At macro-scale, these two approaches are complementary. Their combination in a hybrid framework may help better define the overall impacts [72].

Finally, composite/aggregate indicators (mathematical aggregations of indicators) can give a valuable and accessible overview of the progress and trends of the EU bioeconomy. Presented as an entry point to the underlying data, stakeholders can easily identify high-level trends, make simple comparisons and get a good understanding of the synergies and trade-offs between different components of the bioeconomy [73]. Composite indicators are also powerful communication tools, which can be used to show progress to the media and the public: relevant existing indexes include the SDGs Index [74], the Sustainable Development Index [75], and the Ecological Footprint [76]. However, composite indicators are complex to design and often entail a higher level of value-judgement in their compilation [77]. The EU Bioeconomy Monitoring System will make use of composite indicators, starting from the set of relevant indicators, and summarising key dimensions such as the Strategy Objectives. This will help to generate strong and clear messages for policy makers and other stakeholders.

## Conclusions and next steps

In this paper, we present the approach taken in developing the EU Bioeconomy Monitoring System. Its framework is designed to provide a comprehensive insight into the bioeconomy along the economic, environmental and social dimensions of sustainability, both in the EU as a whole and in Member States. By providing indicators at different levels of aggregation, the monitoring system will provide policy officers with a holistic picture while offering the data to perform in-depth analyses. The framework will also make it possible to look at the impacts of the bioeconomy from different perspectives such as sustainability, ecosystems type and value chain, following the multiple dimensions of the conceptual framework. Finally, the monitoring framework is flexible and may evolve to meet future needs without hampering the capacity to analyse historical changes. This flexibility comes from the multidimensional structure of the framework feeding the overall thematic reporting according to the 2018 EU Bioeconomy Strategy objectives.

Compared to previous efforts to monitor the bioeconomy (see e.g. [58,78,79]), this new framework covers a wider range of perspectives. It takes advantage of the lessons learnt from European initiatives [37,80] and expands them thematically in line with the 2018 EU Bioeconomy Strategy. It also intends to align with international initiatives such as the IBF work on Sustainable Bioeconomy Guidelines [13] and the ISBWG tasks. Advances in EU MSs also inspire the EU monitoring efforts in terms of structure, list of indicators and methods to derive them. A close collaboration with scientists and stakeholders at the EU, national and international level through workshops and bilateral exchanges, ensures the coherence and the usefulness of the EU Bioeconomy Monitoring System.

The EU Bioeconomy Monitoring System is under development following the framework herein described. In an initial mapping of indicators, some gaps have been identified, particularly in the social dimension [31]. Gaps may be due to several reasons, such as the unavailability of data with EU coverage from official statistics or a lack of the appropriate granularity in available statistics in particular for new technologies. For example, the statistics on the chemicals or the plastics and rubber sectors in Eurostat describe both bio-based and fossil-based products and, in the case of drop-in chemicals, it is not possible to make the distinction between the two feedstock sources. In other cases, the data may be available, but only for one point in time. For example, the JRC made an effort to obtain specific data about the bio-based chemical sector through surveys [20] and collection of information from different sources [81], but the level of uncertainty on the numbers retrieved is still high and a stable time series does not yet exist. A long-term solution for a more precise monitoring of these sectors is to add specific codes for bio-based products in the nomenclatures of the databases, as currently developed in the framework of the EU BioMonitor project.

Data gaps can be also observed in innovative and emergent sectors: for example, reliable, robust and temporally consistent information on the algae sector is not available (for both, micro and macroalgae [82]). Algae biomass is considered under different EU collection frameworks but the size of the sector, the confidentiality issues and the still scattered landscape of players hampers the availability of good quality data. Ongoing efforts aim to improve data on the production, on socio-economic and environmental aspects as well as on research and innovation which would be relevant to the bioeconomy monitoring as well as to the Water Framework Directive [83] and the Marine Strategy Framework Directive [84].

The EU framework aims to monitor the impact of the EU bioeconomy in and outside Europe. The latter is complex for some cases and sectors. For example, the impact of imported products – e.g. palm oil, ethanol or meat– can be complex to identify because information about bio-commodities is usually aggregated at country or regional level, hiding the causal links between productive systems and their impacts on biodiversity or greenhouse gasses within a site-specific context. Spatially explicit models can be used to compensate for the lack of data, but these models are not always available or accessible in third countries. Similarly, some indicators are usually not available at the EU and MS level, in particular those corresponding to the status of fish stocks. Techniques to evaluate MSs’ contribution to the depletion or the maintenance of these stocks would be required.

The final set of indicators will need to be balanced across the objectives and criteria; the indicators will also need to be assessed for their quality (see [31,53]). Placeholders will be used where data gaps can be filled neither in a short time nor through proxies. Hopefully, the results of this exercise will also inform further activities for statistical data collections.

Finally, once the final set of indicators is defined, aggregated indicators will be elaborated. The process will again require bringing together scientists and stakeholders to define and parameterize the methods to produce aggregated indicators and represent the results on a publicly available dashboard. This will be the main next step towards the implementation of the EU Bioeconomy Monitoring System.

## Declaration of Competing Interest

The authors declare no conflict of interest. The opinions expressed herein are those of the authors and do not necessarily reflect the views of the European Commission. The scientific output does not imply a policy position of the European Commission.
